# Autism and the broad autism phenotype: familial patterns and intergenerational transmission

**DOI:** 10.1186/1866-1955-5-11

**Published:** 2013-05-02

**Authors:** Noah J Sasson, Kristen SL Lam, Morgan Parlier, Julie L Daniels, Joseph Piven

**Affiliations:** 1 School of Behavioral and Brain Sciences, The University of Texas at Dallas, Richardson, TX 75080, USA; 2 Carolina Institute for Developmental Disabilities, University of North Carolina at Chapel Hill, School of Medicine, Chapel Hill, NC, USA; 3 Department of Epidemiology, University of North Carolina at Chapel Hill, Chapel Hill, NC, USA

**Keywords:** Autism, Broad autism phenotype, Endophenotype, Assessment, Genetics, Personality

## Abstract

**Background:**

Features of the Broad Autism Phenotype (BAP) are disproportionately prevalent in parents of a child with autism, highlighting familial patterns indicative of heritability. It is unclear, however, whether the presence of BAP features in *both* parents confers an increased liability for autism. The current study explores whether the presence of BAP features in two biological parents occurs more frequently in parents of a child with autism relative to comparison parents, whether parental pairs of a child with autism more commonly consist of one or two parents with BAP features, and whether these features are associated with severity of autism behaviors in probands.

**Method:**

Seven hundred eleven parents of a child with an autism spectrum disorder and 981 comparison parents completed the Broad Autism Phenotype Questionnaire. Parents of a child with autism also completed the Social Communication Questionnaire.

**Results:**

Although parental pairs of a child with autism were more likely than comparison parental pairs to have both parents characterized by the presence of the BAP, they more commonly consisted of a single parent with BAP features. The presence of the BAP in parents was associated with the severity of autism behaviors in probands, with the lowest severity occurring for children of parental pairs in which neither parent exhibited a BAP feature. Severity did not differ between children of two affected parents and those of just one.

**Conclusions:**

Collectively, these findings indicate that parental pairs of children with autism frequently consist of a single parent with BAP characteristics and suggest that future studies searching for implicated genes may benefit from a more narrow focus that identifies the transmitting parent. The evidence of intergenerational transmission reported here also provides further confirmation of the high heritability of autism that is unaccounted for by the contribution of de novo mutations currently emphasized in the field of autism genetics.

## Background

Autism is a neurodevelopmental disorder defined by impairments in social interaction, communication, and repetitive and restricted behaviors (American Psychological Association, 1994). Recent advances in genetic research have highlighted rare de novo copy number variants (CNVs) associated with autism (e.g., [1-3]) that occur in higher rates in simplex relative to multiplex families. CNVs, however, are found in only a small minority of autism cases, and no single CNV offers an assurance of autism diagnosis [4]. Indeed, the current focus on CNVs in the study of autism pathogenesis ignores the overwhelming evidence of autism as a heritable condition that dates back to the landmark twin study conducted by Folstein and Rutter [5]. Two recent studies have confirmed the heritability of autism, demonstrating evidence of both concordance [6] and recurrence [7].

De novo CNVs also do not account for the Broad Autism Phenotype (BAP), a term referring to mild but qualitatively similar characteristics of autism [8] that are disproportionately prevalent in parents of a child with an autism spectrum diagnosis [9-13], particularly in multiplex families [14,15]. For example, a recent large-scale study estimates that the prevalence rate of BAP features ranges between 14–23% for parents of a child with autism compared to just 5–9% for a community-based sample of comparison parents [12]. The robust literature on the BAP collectively demonstrates patterns of familial inheritance of autism symptomatology that are unexplained by sporadic mutations. Therefore, while de novo CNVs represent a specific and important genetic mechanism of autism, they do not explain intergenerational inheritance.

Indeed, given that only 10–20% of autism cases currently have an identifiable biological origin [16], any single explanation is unlikely to account for the vast heterogeneity found in autism [17]. Conceptualizing autism characteristics as “fractionable” rather than as the unitary concept required for diagnosis [17,18] may help surmount some of the complexities of genetic heterogeneity. By disaggregating “autism” into dissociable component traits, insights may emerge that facilitate a better understanding of its genetic basis [19]. This approach often focuses on identifying separable subclinical markers of psychiatric conditions present in relatives even without clinical impairment [20], a process that may offer greater promise for specifying genetic vulnerability than broader efforts focusing on intergenerational transmission of qualitative diagnosis [21].

The BAP reflects this approach within autism research, and a large body of literature has accumulated demonstrating subtle autism-related characteristics in family members of an individual with autism (e.g., [9,10,12]). Previous work by our group has isolated three specific behavioral traits of the BAP that correspond to the primary diagnostic domains of autism as defined by DSM IV: (1) social abnormalities, (2) pragmatic language difficulties and (3) rigid personality and a desire for sameness. These BAP traits may inform genetic liability by examining their patterns of segregation within families of an individual with autism (e.g., [11]) as well as their higher rates in multiple relative to single-incident families (e.g., [15]), which may reflect an effect of increased genetic loading.

While previous work by our group has demonstrated that all three BAP features are more prevalent in individual parents of a child with autism relative to individual comparison parents [12,22], a number of issues about familial patterns remain unclear and require large-scale samples for study. For example, it is uncertain whether the presence of BAP features in *both* parents confers an increased liability for autism. If found, such evidence may indicate that autism in part arises from distinct genetic contributions from each parent, such that transmission from one parent alone may produce only the BAP or its individual features, while transmission from both parents in co-dominant fashion increases the risk of autism. In contrast, evidence that the presence of two affected parents confers little to no additional risk for autism beyond the presence of a single affected parent may indicate significant specificity of genetic liability. If occurring with high penetrance, such a finding could benefit genetic investigations by narrowing the focus of study by identifying the transmitting parent. Thus, the examination of co-occurrence of BAP traits within both parents of children with autism helps address whether genetic liability for autism is better characterized as transmitted through one (uni-lineal) or both (bi-lineal) parents.

The current study was designed to provide inferences about potential genetic mechanisms by examining patterns of the autism phenotype within families of an individual with autism. Specifically, the primary aim of this study is to explore whether the presence of BAP features within parents, both individually and jointly, is higher in families of a child with autism and whether these features are associated with severity of autism behaviors in probands. We first address this issue by determining if the presence of BAP features in both biological parents occurs more frequently in parents of a child with autism relative to comparison parents. Such a finding would suggest that co-occurrence of BAP features within both parents increases the genetic liability of autism. Next, we examine the degree to which intergenerational transmission of autism is characterized as uni-lineal or bi-lineal. Uni-lineal transmission would be suggested by BAP features occurring more commonly for just one parent of a child with autism, while bi-lineal transmission would be suggested by BAP features occurring more commonly for both parents.

Previous work has linked autism-related characteristics between parents and children in the general population [23], yet little is known about whether the presence of BAP features, both independently and in combination (i.e., a “dose effect”), is associated with severity of autism behaviors in probands. To address this issue, we first compare the severity of autism behaviors between children of parents who do and do not have each of the three BAP features. We also explore evidence of “dose” effects by assessing (1) whether individual parents with multiple BAP features have children with greater severity of autism behaviors than parents with a single BAP feature and (2) whether parental pairs in which both parents exhibit BAP features have children with greater severity of autism behaviors relative to those in which only one parent is affected. Evidence of each would suggest that the presence of multiple BAP features in parents, both as individuals and in combination, confers an increased genetic liability to autism.

Finally we conduct an exploratory analysis aimed at determining whether the presence of both social (i.e., aloof personality and/or pragmatic language abnormalities) and nonsocial (i.e., rigidity) BAP traits within one or both parents increases autism severity in children relative to those with just social *or* nonsocial traits and those with neither. Reorganizing conceptualization of the BAP into bifurcated social and nonsocial traits aligns with the recent diagnostic changes of autism in the DSM5. Thus this analysis serves two purposes: to examine whether co-occurrence of distinct autism-related features within parents increases the severity of autism behaviors in children and as a proof of concept for organizing the BAP into two rather than three separable components.

## Methods

### Sample

Seven hundred eleven parents of a child with an autism spectrum disorder and 981 comparison parents participated in this study. Parents of a child with autism were recruited from the Autism Registry of the Carolina Institute for Developmental Disabilities (CIDD), which consists of approximately 5,000 nuclear families in North Carolina that include an individual with a diagnosis on the autism spectrum, as determined by a licensed clinician with expertise in autism diagnosis and assessment, and have consented to be contacted regarding possible participation in research studies on autism. Inclusion criteria required that these parents be between 18 and 65 years, primarily English speaking, and the biological parent of a child 18 years old or younger who had been clinically evaluated by TEACCH (Treatment and Education of Autistic and related Communication-handicapped Children) or during participation in prior CIDD research studies. Comparison parents were contacted via community mailings to families identified through North Carolina birth records who had signed a consent form to be contacted about research opportunities. Neither comparison parents nor their children were screened for developmental disorders or other diagnoses, and thus this group should reflect the distribution of BAP characteristics within the local community population rather than a case control group matched to the group of parents of a child with autism. The two groups did not differ on gender or number of biological children, but did on age and education, and thus these variables were co-varied in all ANOVAs comparing the groups. Further information about the ascertainment and characterization of this sample can be found in Sasson et al. [12]. The IRB at the University of North Carolina at Chapel Hill approved the protocol for this study, and all participants provided informed consent.

### Measures

#### Assessment of the broad autism phenotype

BAP features in parents were assessed with the Broad Autism Phenotype Questionnaire (BAPQ) [22]. The BAPQ is a self- and informant-report measure consisting of 36 items derived from gold-standard clinical interview methods [24,25]. Each of the three 12-item subscales of the BAPQ corresponds to one of the three primary impairments of autism, social abnormalities, pragmatic language difficulties and rigid personality, and thus enables the quantification of separable BAP features. Items are rated on a one to six scale ranging from “very rarely” to “very often.” Recent psychometric evaluation using exploratory factor analysis confirms that the BAPQ consists of a three-component structure corresponding to the intended aloof, pragmatic language and rigidity subscales, with high internal consistency for each [12]. The BAPQ includes separate cutoff values that maximize sensitivity and specificity for identifying the BAP [12,22].

Participants were asked to complete the self-report version of the BAPQ as well as the informant version about their child’s other biological parent. They were instructed to work independently, guess when unsure of an answer, and respond to items in reference to (1) their behavior during most of their adult lives rather than just a specific period in time and (2) as it relates to interactions with most people instead of a close relationship. The BAPQ was presented to them as “The Personality and Preferences Questionnaire” in order to reduce bias associated with beliefs about autism.

#### Assessment of autism characteristics in children

Parents of a child with autism, but not comparison parents, completed the Social Communication Questionnaire (SCQ) [26] for their child. The SCQ is a parent-completed instrument developed based upon the Autism Diagnostic Interview-Revised (ADI-R) [27], which is well validated for children older than 2 years of age [28]. No child with autism in our sample was under 3 years of age at the time of testing. Scores can range from 0 to 39, with higher scores indicating greater severity of autism behaviors. The SCQ consists of three subscales, social (15 items), communication (13 items), and restricted & repetitive (8 items), which relate to the three core domains of autism. The SCQ was used in this study because it offered an efficient tool for measuring autism severity in a large sample without face-to-face contact. Parents who reported more than one child with autism (12.1%) were asked to complete the SCQ for the child they believed to be most severely affected.

### Data analysis

Parents were first categorized as “present” or “absent” on the composite BAP and on each of the three BAP features based upon whether their BAPQ values exceeded the cutoff values reported in Sasson et al. [12]. These cutoff values were derived by identifying composite and subscale BAPQ scores of 1.5 SD above the mean for men and women in the comparison sample, and subsequent analysis demonstrated that they maximize specificity (i.e., they virtually eliminate false positives) when evaluated in reference to BAP status obtained via direct clinical assessments. The averages of the subject and informant versions of the BAPQ were used in this study in order to mitigate bias, and thus only instances in which both parents completed each version were examined (73.4% of the sample). BAP prevalence rates using the subject-informant average are comparable to those generated by using the subject or informant version alone [12].

To assess whether co-occurrence of BAP features is more common for parental pairs of a child with autism relative to comparison parental pairs, the percentage of parental pairs in which 0, 1 or 2 parents were “present” for the BAP composite and at least one BAP feature was compared between groups using chi-square analyses. We then used a chi-square test to compare the proportion of parental pairs of a child with autism in which both are positive for BAP features compared to the proportion in which such traits occur for only one parent.

To examine whether the BAP in parents is associated with severity of autism behaviors in probands, parents of a child with autism characterized as BAP “present” or BAP “absent” and the children’s overall and subscale SCQ scores were compared between groups. This was done for the BAP composite as well as on each of the three BAPQ subscales using independent t-tests. We then assessed whether multiple BAP features in parents is associated with greater autism severity in children (i.e., a “dose” effect) by comparing SCQ scores between children of parents differing in the number of present BAP features (0, 1, 2 or 3) using an ANOVA. Similarly, we examined whether a dose effect occurred for parents by using an ANOVA to determine if SCQ scores differed between children of 0, 1 or 2 parents positive on at least one BAP feature. Finally, we use an ANOVA to explore whether SCQ scores are higher in children of parental pairs characterized by both social (i.e., aloof and/or pragmatic language difficulties) and nonsocial (i.e., rigidity) BAP features relative to those with only one of these features and those with none at all.

## Results

The proportion of parental pairs in which neither, one or both were positive for the BAP composite, at least one BAP feature, and any two BAP features, can be viewed in Table 1. Parental pairs of a child with autism were significantly more likely than comparison parental pairs to have both parents positive for the composite BAP and at least one BAP feature. Only a small proportion of parental pairs of a child with autism (4.3%) and comparison parental pairs (1.6%) were comprised of both parents positive on the BAP composite, though this was a significant difference [*X*^2^ (1, 562) = 3.75, *p* = 0.05]. The proportion of parental pairs in which both parents exhibited at least one BAP feature was 15.1% for those of a child with autism compared to 5.3% of comparison parental pairs, also a significant difference [*X*^2^ (1, 562) = 15.10, *p* < 0.001]. Parental pairs of a child with autism were significantly more likely to consist of a single parent being positive on the BAP composite (31.7%) compared to both parents being positive on the BAP composite (4.3%) [*X*^2^ (1, 186) = 3.88, *p* = 0.05] and also significantly more likely to consist of a single parent being positive on any one BAP feature (38.2%) compared to both parents being positive on any one BAP feature (15.1%) [*X*^2^ (1, 186) = 20.82, *p* < 0.001]. Of the parental pairs of a child with autism who consisted of a single parent positive on any one BAP feature, exactly half of the affected parents were fathers and half were mothers.

**Table 1:** Proportion of parental pairs in which neither, one or both parents were positive on the overall BAP composite, any one BAP feature and any two BAP features

	**Parental pairs of a child with autism**	**Comparison parental pairs**	***p*-value**
*No. of parents positive on BAP composite*			
Neither	64.0%	85.9%	<0.001
One	31.7%	12.5%	<0.001
Both	4.3%	1.6%	0.05
*No. of parents positive on any one BAP Feature*			
Neither	46.2%	68.9%	<0.001
One	38.7%	25.8%	0.002
Both	15.1%	5.3%	<0.001
*No. of parents positive on any two BAPs Features*			
Neither	75.3%	93.1%	<0.001
One	22.6%	6.6%	<0.001
Both	2.2%	0.3%	0.03

Composite SCQ scores for children with autism were normally distributed, with acceptable levels of skew and kurtosis and a non-significant result on the Shapiro-Wilk W test. SCQ scores did not differ between male (*n* = 268; M = 21.76, SD = 6.54) and female (*n* = 77; M = 21.87, SD = 6.65) children (*p* = 0.90). “BAP present” (i.e., those whose composite BAPQ scores exceeded the averaged subject-informant cutoff scores) and “BAP absent” parents of a child with autism did not differ on any demographic variable and thus these demographic variables were not included in analyses. SCQ total and subscale scores for children of parents “present” and “absent” for the BAP composite and each BAP feature can be viewed in Table 2. “BAP present” parents had children with higher SCQ scores (M = 23.49, SD = 6.30) than “BAP absent” parents (M = 21.52, SD = 6.56) [t (343) = 1.93, *p* = 0.05]. This pattern differed by subscale. Whereas “aloof present” parents had children with higher SCQ scores (M = 23.59, SD = 6.44) than “aloof absent” parents (M = 21.28, SD = 6.52) [t (343) = 2.46, *p* = 0.01], a trend only emerged for higher SCQ scores in children of “pragmatic language present” parents relative to their “absent” counterparts (*p* = 0.07), and SCQ scores did not significantly differ between “rigidity present” and “rigidity absent” parents (*p* = 0.48). Proband gender did not differ significantly between “present” and “absent” parents on the BAP composite or on any BAP feature.

**Table 2:** SCQ total and subscale scores for children with autism of parents “present” and “absent” for the BAP composite and each BAP feature

	**BAP composite**			**BAP aloof**			**BAP pragmatic language**			**BAP rigidity**		
	**Present**	**Absent**	***p***	**Present**	**Absent**	***p***	**Present**	**Absent**	***p***	**Present**	**Absent**	***p***
*SCQ Scale*												
Total	23.49 (6.30)	21.52 (6.56)	**0.05**	23.59 (6.44)	21.28 (6.52)	**0.01**	23.16 (6.91)	21.48 (6.45)	0.07	22.38 (6.73)	21.68 (6.53)	0.48
Social	8.47 (3.40)	7.47 (3.51)	**0.02**	8.43 (3.58)	7.51 (3.47)	**0.04**	8.73 (3.43)	7.45 (3.49)	**<0.01**	8.21 (3.40)	7.58 (3.52)	0.19
Communication	7.13 (2.49)	6.61 (2.50)	0.10	7.38 (2.35)	6.57 (2.52)	**0.01**	6.95 (2.78)	6.67 (2.45)	0.61	7.00 (2.70)	6.67 (2.47)	0.24
Restricted & repetitive	5.97 (1.87)	5.77 (1.88)	0.39	6.05 (1.88)	5.76 (1.88)	0.34	6.06 (1.80)	5.76 (1.89)	0.12	5.83 (1.78)	5.81 (1.90)	0.74

Do parents of a child with autism exhibiting more BAP features have children with higher SCQ scores? Parents with 0, 1, 2 and 3 BAP features did not differ on any demographic variable, and thus they were not included in the ANOVA. SCQ scores of children differed depending upon the number of BAP features exhibited by their parents [F (3, 341) = 3.44, *p* = 0.017]. Post-hoc Tukey tests revealed that this was driven by significantly higher SCQ scores in children of parents with all three BAP features (M = 28.00, SD = 7.12) relative to those with two (M = 22.21, SD = 6.35), one (M = 21.99, SD = 6.12) or no BAP features (M = 21.37, SD = 6.60), while SCQ scores did not differ between children of parents with 0, 1 or 2 features.

To further address issues of uni- versus bilineal transmission, we also examined whether having more than one affected parent is associated with higher SCQ scores in children. SCQ scores differed depending on whether 0, 1 or 2 parents had at least one BAP feature [F (2, 169) = 3.81, *p* = 0.02]. Post-hoc Tukey tests revealed that this was driven by significantly higher SCQ scores for children of one (M = 22.86, SD = 6.07) or two (M = 23.52, SD = 7.10) parents with a BAP feature relative to children of parents in which neither had a BAP feature (M = 20.34, SD = 6.53), while SCQ scores did not differ between children of one or two parents with a BAP feature. Similarly, SCQ scores significantly differed between parental pairs with both social and nonsocial BAP features (M = 23.15, SD = 6.79), those with just one of these features (M = 22.62, SD = 5.84) and those with neither of these features (M = 19.87, SD = 6.61) [F (2, 169) = 4.54, *p* = 0.01]. Post-hoc Tukey tests determined that this occurred because of significantly lower SCQ scores in parental pairs with neither social nor nonsocial features relative to those with both features (*p* = 0.04) and to those with just one (*p* = 0.04), with no difference in SCQ scores between those with both features compared to those with just one (*p* = 0.92).

## Discussion

The present study employs a large sample to explore the relationship between BAP features in parents and the prevalence and severity of autism in children. While previous research has reliably demonstrated that biological parents of children with autism exhibit BAP traits to a greater degree than comparison parents [12], the present study focuses on parental pairs to explore whether genetic liability for autism increases when both parents exhibit BAP features.

Parental pairs of a child with autism were significantly more likely than comparison parental pairs to consist of two BAP-“positive” parents, yet this proportion was relatively small. Just 4.3% of parental pairs of a child with autism consisted of both parents positive on the BAP composite and 15.1% consisted of both exhibiting at least one BAP feature. Though higher than in comparison parental pairs, the low percentage of two affected parents suggests that only a small subgroup presents evidence consistent with bilineal transmission of autism. In contrast, a significantly larger proportion of parental pairs of a child with autism, approximately 1/3rd, consisted of only one parent positive for the composite BAP, and nearly 40% consisted of just one parent exhibiting at least one BAP feature. Thus, evidence from this study suggests that intergenerational transmission likely occurs in at least a 1/3rd of families and genetic involvement is not exclusively de novo.

We also examined whether the presence of BAP traits in parents of children with autism is associated with the severity of their child’s autism behaviors. Children of BAP “present” parents were characterized by higher SCQ scores than those of BAP “absent” parents. This link between parental characteristics and child autism severity is consistent with theories of multifactorial inheritance [29] and suggests that the BAP in parents is not just related to qualitative diagnosis of autism in children, but also may be associated with quantitative expression of autism behaviors. This pattern differed for specific BAP traits, however, with parents exhibiting aloof personality having children with higher SCQ scores than those without this BAP feature, while no difference emerged for SCQ scores between parents with and without rigid personality. Such a distinction suggests that separable BAP features may relate differentially to genetic liability of autism. Additionally, parents with aloof and pragmatic language traits had children with higher scores on the social, but not the rigidity, subscale of the SCQ, suggesting a level of intergenerational consistency in social aspects of the autism phenotype that may warrant further investigation.

Further, although parents with all three BAP features had children with the highest SCQ scores, no difference in SCQ scores occurred between children of parents with zero, one or two BAP features. Similarly, while SCQ scores were lowest for children of parental pairs in which neither parent was BAP-“positive,” there was no difference in SCQ scores between children of parental pairs with two BAP-“positive” parents and those with one. An analogous pattern was also found when the BAPQ subscales were reorganized to align with DSM5 criteria, with SCQ scores not differing between parental pairs with both social and nonsocial features compared to those exhibiting just one of these features. Thus, there is very little evidence in the current study that having both parents with BAP traits increases the severity of autism behaviors in children above and beyond the severity conferred from having one affected parent. Rather, severity appears most predictive by the presence of any single BAP feature in parents relative to parents with no BAP features, with little added risk provided by the presence of additional features.

Collectively, findings from the present study suggest that a significant proportion of families of a child with autism consist of a single parent with BAP features. If penetrance is assumed to be high, future genetic studies may benefit from a stronger focus on the identification and examination of the transmitting parent. Such an approach could lead to more refined methods for locating implicated genes. For example, studies could search for shared CNVs between probands and affected parents or pursue linkage analysis that identifies parents with BAP features as affected and those without BAP features as ‘unknown.’ Currently, relatives in family studies are typically classified as unaffected if they do not have an autism spectrum disorder. The addition of BAP information affords the opportunity to include a third classification of BAP status that may increase the power of the study. Given the efficiency of the instruments available (e.g., BAPQ, SCQ) that can be administered remotely (e.g., over the Internet), the possibility now exists for patterns of familial transmission to be examined in even larger scale studies in which ascertainment is clearly incorporated into the model.

These findings should be interpreted within the context of several limitations. First, despite using a large sample, relatively few parental pairs consisted of two BAP-“positive” parents, thereby limiting power to detect patterns specific to these families. It is possible that BAP-affected parents are more likely to decline participation than parents without BAP traits, which may have reduced the number of parental pairs of a child with autism ascertained with two BAP-affected parents. Future epidemiological and genetic studies attempting to address questions of bilineality in greater depth should consider examining an even larger sample. Second, all categorizations of BAP status were derived from the average of self- and informant-reported versions of the BAPQ. This was done in order to minimize bias that could have occurred from reliance on only one of the versions, but this BAP status may differ from best estimates generated from in vivo clinical assessments. Depending on research goals, future studies may choose to categorize BAP status based upon one of the versions, or even the higher of the two (see [12] for a larger discussion of this point). Fourth, we did not assess the clinical status of parents. Although autism severity was highest for children of parents with all three BAP features, we were unable to determine whether these parents may have met criteria for an autism spectrum disorder. Finally, though the current study underscores the relevance of familial patterns of autism-related traits to the search for genetic mechanisms, explicit examination of these relationships will require large-scale genetic analyses.

## Conclusions

These limitations notwithstanding, this study offers a strong foundation for future research investigating the etiology of autism. By linking prevalence and severity of autism in children to BAP traits in parents, the current study demonstrates that important genetic mechanisms of autism extend beyond de novo mutations and suggests that patterns of transmission may be profitably pursued by incorporating the rich information of familial inheritance provided by the BAP. Segregating autism-related traits in families through the examination of the BAP may be useful in identifying implicated genes, at least in a subset of families, and thus future studies aimed at specifying the genetic underpinnings of autism are encouraged to consider this information in their investigations.

## Abbreviations

ANOVA: Analysis of variance; BAP: Broad autism phenotype; BAPQ: Broad autism phenotype questionnaire; CIDD: Carolina Institute for Developmental Disabilities; CNVs: Copy number variants; DSMIV: Diagnostic and Statistical Manual of Mental Disorders, 4th edition; DSM5: Diagnostic and Statistical Manual of Mental Disorders, 5th edition; SCQ: Social communication questionnaire

## Competing interests

The authors declare that they have no competing interests.

## Authors’ contributions

NJS performed data analysis and interpretation, and prepared the manuscript; KSLL performed data analysis and interpretation; MP participated in data collection and interpretation; JD participated in the design of the study, oversaw data collection and aided manuscript preparation; JP conceived of the study, participated in design and coordination, and supported data interpretation and manuscript preparation. All authors read and approved the final version of the manuscript.
